# Clove, Cinnamon, and Peppermint Essential Oils as Antibiofilm Agents Against *Alicyclobacillus acidoterrestris*

**DOI:** 10.3390/molecules30112312

**Published:** 2025-05-24

**Authors:** Agnieszka Tyfa, Alina Kunicka-Styczyńska, Magdalena Molska, Radosław Michał Gruska, Andrzej Baryga

**Affiliations:** 1 Institute of Agricultural and Food Biotechnology—State Research Institute (IBPRS-PIB), Food Quality Department, Al. Marszałka J. Piłsudskiego 84, 92-202 Lodz, Poland; agnieszka.tyfa@ibprs.pl; 2 Department of Sugar Industry and Food Safety Management, Faculty of Biotechnology and Food Sciences, Lodz University of Technology, Wólczańska 171/173, 90-530 Lodz, Poland; magdalena.molska@p.lodz.pl (M.M.); radoslaw.gruska@p.lodz.pl (R.M.G.); andrzej.baryga@p.lodz.pl (A.B.)

**Keywords:** *Alicyclobacillus acidoterrestris*, bacterial biofilm, essential oils, antibiofilm agents

## Abstract

*Alicyclobacillus acidoterrestris*, an acidothermophilic bacterium, is one of the main contaminants in the fruit industry due to its high tolerance to environmental stress and ability to form biofilms. As conventional methods of biofilm elimination may be ineffective, there is a need for safe and sustainable methods for *A. acidoterrestris* management. The objective of the study was to evaluate the antibiofilm activity of commercial essential oils derived from clove (*Syzygium aromaticum* (L.) Merr. & Perry), cinnamon (*Cinnamomum zeylanicum* Blume), and peppermint (*Mentha piperita* (L.) Huds.) against *A. acidoterrestris*. The effect of the essential oils on the mature biofilms of fifteen environmental *A. acidoterrestris* isolates subjected to variable pH values (3.0, 4.0, and 5.5), temperatures (25, 37, and 44 °C), and essential oil concentrations (0.5 MIC, MIC, and 2 MIC compared to planktonic cells) was studied. The essential oils showed significant antibiofilm activity against *A. acidoterrestris* isolates, with the efficiency of biofilm eradication depending on the strain and environmental parameters such as acidity, culture temperature, type, and concentration of essential oil. The greatest antibiofilm potential was observed for clove essential oil regardless of oil concentrations and environmental conditions. Cinnamon oil exhibited lower activity against the tested *A. acidoterrestris* strains. Peppermint essential oil showed the weakest antibiofilm ability and did not completely eradicate any strain biofilm of the tested strains. Clove and cinnamon essential oils have the potential to be effective alternatives to synthetic disinfectants directed against *A. acidoterrestris* grown in the form of biofilms.

## 1. Introduction

In industrial environments, including the food industry, bacteria rarely exist in the form of planktonic cells, and the colonization of abiotic surfaces is strictly connected with biofilm formation. Bacterial biofilms are a source of cells spreading throughout production processes, which usually results in the contamination of final food products and potential economic losses as well as a threat for human health. The fruit industry has been struggling with *Alicyclobacillus acidoterrestris* contamination for decades [1,2,3,4,5]. *A. acidoterrestris* are Gram-positive, spore-forming, aerobic bacteria that were first isolated in 1982 from spoiled pasteurized apple juice [1]. They are able to grow in highly acidic environments (pH 2.0–6.0) and at elevated temperatures (20–60 °C) [1,2,6,7,8]. Their presence in the industry has been confirmed worldwide in various food products, particularly fruit juices. According to a 1998 survey of Research by the National Food Processors Association (NFPA), *Alicyclobacillus* spp. were responsible for 35% of fruit juice contaminants in the fruit industry [9,10]. *A. acidoterrestis* were isolated from apple, blackcurrant, cherry, chokeberry, grapefruit, mango, orange, passion fruit, pineapple, peach, raspberry, strawberry, tomato juices, surface of fruits (apple, kiwi, pear), coconut milk, and ingredients for beverage production [2,11,12,13,14,15,16,17,18,19,20,21,22,23]. Products spoilage with *A. acidoterrestris* originates from the transmission of bacterial cells or spores from contaminated fruit to subsequent production stages. The ability of *Alicyclobacillus* spp. to withstand and grow in thermophilic and acidophilic environments makes them resistant to pasteurization processes. Moreover, heat treatment activates their spores, causing germination, growth, and the colonization of production environments in the form of biofilms.

Biofilms are multicellular communities of microorganisms formed when the individual cells attach to biotic or abiotic surfaces, subsequently growing to form microcolonies [24,25]. The cells within the biofilm are well protected against changing environmental conditions thanks to the polymer matrix produced, which consists mainly of polysaccharides, proteins, phospholipids, and nucleic acids. During maturation, some of the biofilms decompose and disperse, leading to the contamination of other surfaces or the new biofilms [26,27].

*A. acidoterrestris* adhesion and biofilm formation on abiotic surfaces, e.g., glass, nylon, PVC, polystyrene, rubber, and stainless steel surfaces, has been confirmed [28,29,30,31]. Additionally, it has been reported that external environmental conditions can intensify adhesion and biofilm development [30,31,32,33,34]. Established biofilms are generally difficult to remove due to their complex composition and structure, which results in a low susceptibility to the recommended disinfectants. Standard industrial methods for the inactivation of contaminating microorganisms are often insufficient. Moreover, consumers expect food products to be of high quality, to maintain their original properties, and to have a long shelf life without the addition of synthetic preservatives. Therefore, the use of natural plant substances as antibiofilm agents has gained the attention of scientists and producers.

Essential oils (EOs) are considered as a natural alternative to synthetic preservatives that can improve the safety and shelf life of food products. Essential oils are secondary plant metabolites with a complex chemical composition that varies depending on the oil, the part of the plant, the season of collection, and the extraction method. EOs serve as a source of many compounds with biological activity, and depending on their qualitative and quantitative composition, they can exhibit a variety of bioactive properties [35,36,37]. Among the active compounds of EOs, phenols, aldehydes, terpene alcohols, ethers, and ketones express proven antimicrobial activity, with the highest activity mainly associated with phenols, particularly carvacrol, eugenol, and thymol [38,39,40,41].

It has been demonstrated that the addition of essential oils can reduce the heat treatment temperature of fruit juices. In addition, essential oils have been proven to be effective against both planktonic bacteria and biofilms [42,43,44,45]. Several oils and their components were tested as natural agents inhibiting both the growth and biofilm formation of microorganisms contaminating the food industry. EOs are substances with historically documented, long, and well-established use in food and cosmetics. According to the FDA, the majority of essential oils, including cinnamon, clove, and peppermint, which are used in food as flavor ingredients, are Generally Recognized as Safe [46,47]. The direct application of these oils as food components is limited to low concentrations due to their organoleptic features, which is also reflected in consumer acceptance. The application of essential oils as antibiofilm substances requires higher concentrations, which is associated with increased toxicity. Considering the oils’ potential effectiveness against bacterial biofilms, their implementation as disinfectants applied onto abiotic surfaces in the food industry seems rational for safety reasons. Cinnamon essential oil inhibits the growth and shows antibiofilm effect against foodborne pathogens [45,48,49] as well as preventing bacterial adhesion to stainless steel and polystyrene [49,50]. Antibiofilm action was observed towards *Escherichia coli* O157:H7, *Listeria monocytogenes*, and *Staphylococcus aureus* for one of the most active components of cinnamon oil, eugenol [51,52,53]. Similarly, cinnamaldehyde, another strong component of cinnamon oil, influences bacterial biofilm formation and reduces the number of sessile cells [50,54]. Moreover, cinnamon and clove essential oils may serve as bioactive compounds in packaging that supports the control of microbial growth in fruit industry [55,56]. The growth of *A. acidoterrestris* was inhibited in the presence of lemon essential oil and lemon extract [57,58]. Several oil components, such as citral, limonene, eugenol, and cinnamaldehyde, are known to limit the propagation of these acidotermophilic bacteria as well [59,60]. Peppermint oil is a well-known substance that improves food quality [61]. In addition, peppermint oil exhibits antimicrobial properties against many microorganisms, including foodborne pathogens and other food spoilage microorganisms together with their biofilms [62,63,64,65]. The activity of this oil and its main components towards Gram-positive spore-forming *Bacillus subtilis* and *Bacillus cereus* seems promising against *A. acidoterrestris* as well [66,67]. Our previous study proved the effectiveness of clove essential oil against *A. acidoterrestris* biofilms formed on glass and polyvinyl chloride surfaces [32]. Given the potential use of essential oils as antibiofilm agents in the juice industry, their activity towards *A. acidoterrestris* environmental isolates requires further investigation.

The aim of the study was to evaluate the antibiofilm activity of clove (*Syzygium aromaticum* (L.) Merr. & Perry), cinnamon (*Cinnamomum zeylanicum* Blume), and peppermint (*Mentha piperita* (L.) Huds.) essential oils against *A. acidoterrestris* under variable environmental conditions. To optimize the oils’ effectiveness, their effect on the biofilms formed by 15 environmental *A. acidoterrestris* isolates under different acidity and temperature conditions was evaluated.

## 2. Results

### 2.1. Essential Oils’ Chemical Composition

The chemical compositions of clove, cinnamon, and peppermint essential oils are presented in Table 1. Fourteen components representing 99.06% of the total constituents were found in clove essential oil, with eugenol (86.99%) and β-caryophyllene (8.76%) dominating. Based on the GC-MS analysis of cinnamon essential oil, 14 components were identified, representing 99.40% of total constituents. The predominant components were sesquiterpenes and aldehydes, with trans-cinnamaldehyde (72.49%), β-caryophyllene (8.12%), linalool (6.59%), and eugenol (5.17%) being the main ones. The main components of peppermint essential oil were (−)-menthol (46.41%), (−)-menthone (22.46%), and 1,8-cineole (6.00%). In total, 17 components (96.85%) were identified, with monoterpenes, sesquiterpenes, and alcohols predominating. The chemical profiles of the essential oils investigated were in agreement with the literature data and met the requirements and standards of European Pharmacopeia in regard to the main constituents that are the most important for the retaining of the oil identity [68]. Minor differences were noted for clove essential oil, as it lacked eugenyl acetate, the content of which in this oil is often in the range of 0.3–21.3% [69,70].

### 2.2. Essential Oils’ Activity Against A. acidoterrestris Plaktonic Cells 

The environmental strains of *A. acidoterrestris* showed a high sensitivity to cinnamon oil. The minimum oil concentration at which growth inhibition was observed (MIC) was lower than 0.05% and the minimum bactericidal concentration (MBC) was 0.05% for four strains (055, 056, 057, and 063). The growth of the reference strain *A. acidoterrestris* DSM 3922, as well as isolates 008 and 026, was inhibited in the presence of cinnamon oil at a concentration of 3.0%. The remaining isolates exhibited a higher resistance to this oil, and the determined MIC values exceeded 5.0% (Table 2). The MIC values of clove oil against *A. acidoterrestris* DSM 3922 and isolates 055, 056, 057, and 063 were equal to 0.05% (MBC 0.06%). The other strains tested showed low sensitivity to clove oil, with MICs above 5.0% (Table 2). The reference strain and three isolates (056, 057, and 063) displayed a high susceptibility to peppermint oil (MIC 0.05%), whereas for the remaining isolates, the MIC values did not exceed 3.0%. The high sensitivity of isolates 056, 057, and 063 to essential oils was confirmed by the low MBC values of clove (0.06%), peppermint (0.06%), and cinnamon oils (0.05%).

### 2.3. Biofilm Formation and Eradication with Essential Oils

The activity of clove, cinnamon, and peppermint essential oils against the mature biofilms of sixteen *A. acidoterrestris* strains was investigated under various environmental conditions. For each bacterial strain, the following variables were tested: pH (3.0, 4.0, and 5.5), temperature (25, 37, and 44 °C), and essential oil concentrations (0.5 MIC, MIC, and 2 MIC). This resulted in 81 variants of culturing for each strain and 1296 variants in total for the research. A principal component analysis (PCA) was used to analyze similarities in the sensitivity of *A. acidoterrestris* biofilms to the oils, resulting in their inhibition or eradication.

**Figure 1:**
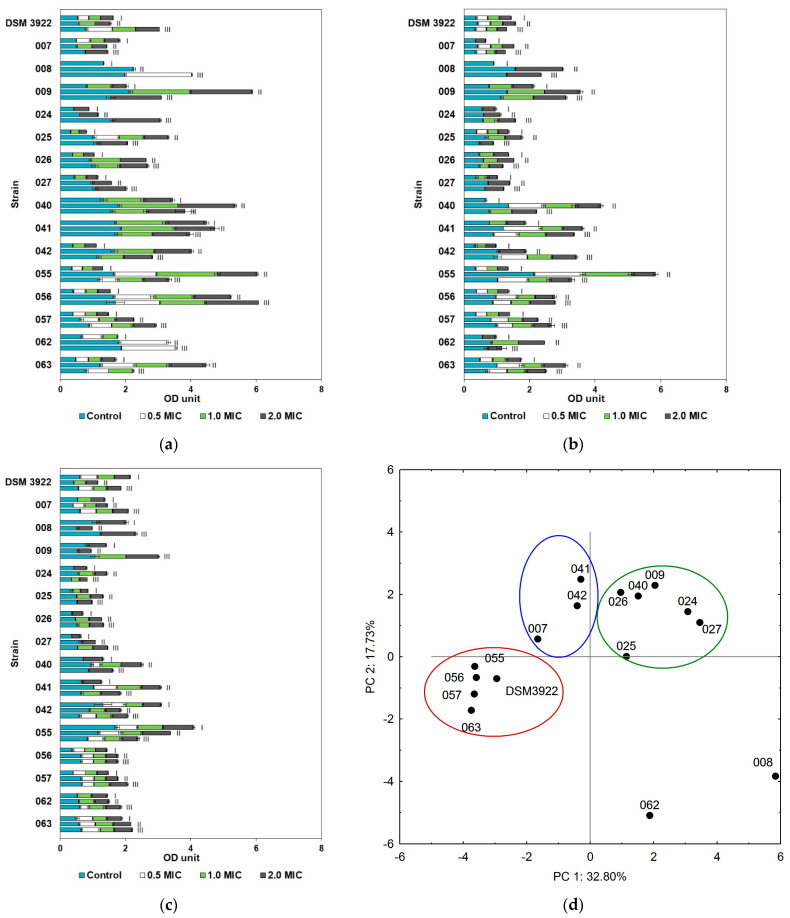
*A. acidoterrestris* biofilm formation in the presence of clove essential oil at pH 3.0 (**a**), pH 4.0 (**b**), and pH 5.5 (**c**) and the temperatures of 25 °C (I), 37 °C (II), and 44 °C (III); colors represent biofilms grown with essential oil (EO) concentration: light blue—without oil, white—0.5 MIC, light green—MIC, dark grey—2.0 MIC. (**d**) PCA analysis of *A. acidoterrestris* biofilm eradication with clove oil (factors describe 50.53% total variance); blue, green and red circles represent clusters of strains with similar abilities.

**Figure 2:**
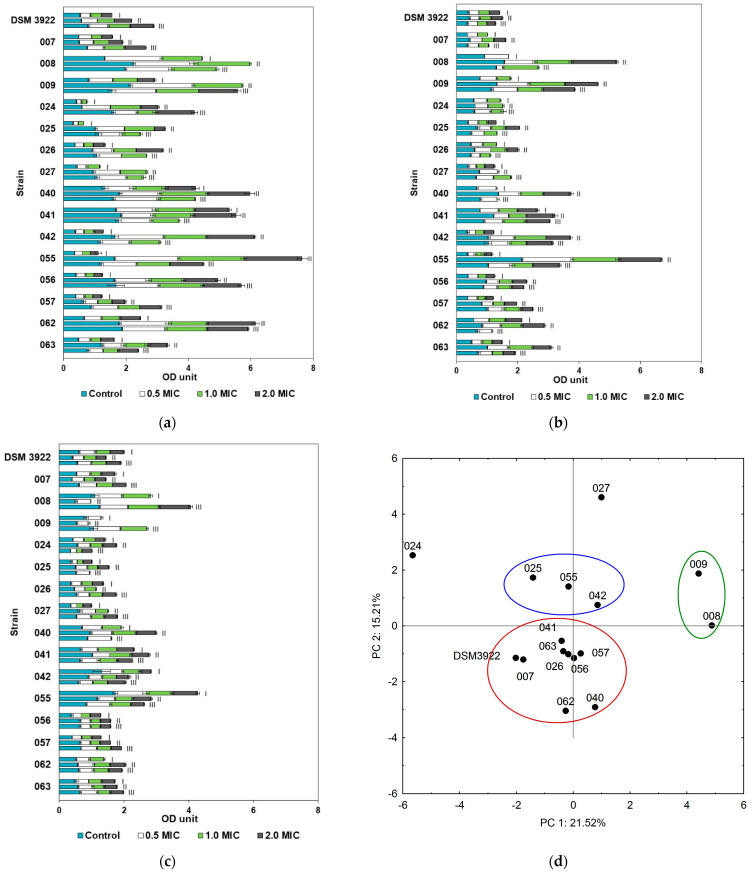
*A. acidoterrestris* biofilm formation in the presence of cinnamon essential oil at pH 3.0 (**a**), pH 4.0 (**b**), and pH 5.5 (**c**) and the temperatures of 25 °C (I), 37 °C (II), and 44 °C (III); colors represent biofilms grown with essential oil (EO) concentration: light blue—without oil, white—0.5 MIC, light green—MIC, dark grey—2.0 MIC. (**d**) PCA analysis of *A. acidoterrestris* biofilm eradication with cinnamon (factors describe 36.73% total variance); blue, green and red circles represent clusters of strains with similar abilities.

**Figure 3:**
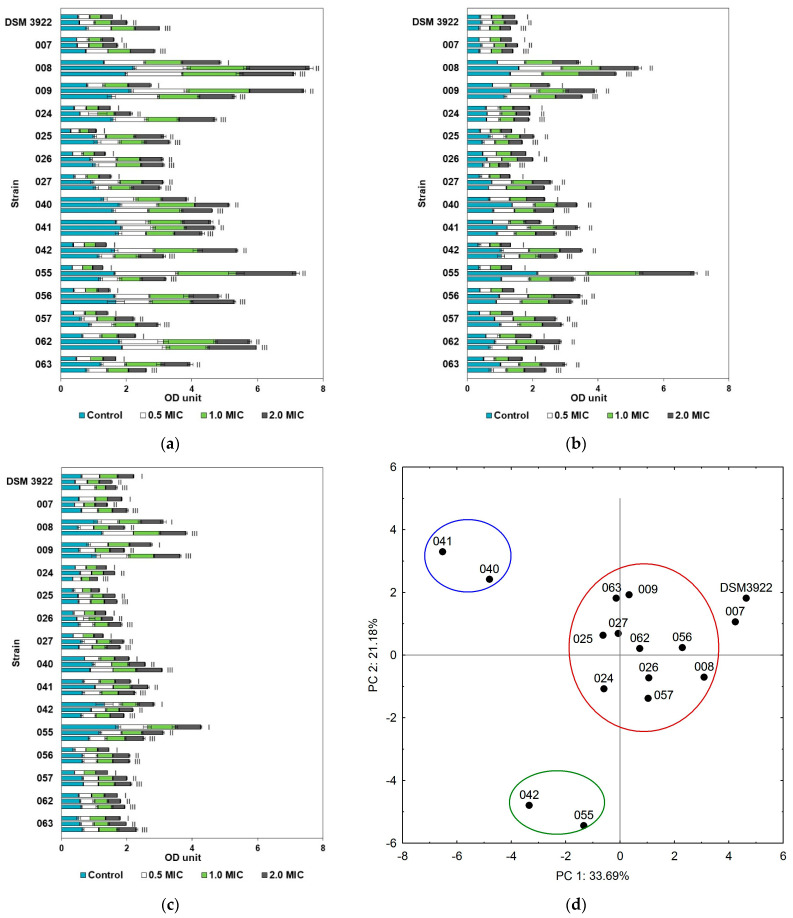
*A. acidoterrestris* biofilm formation in the presence of peppermint essential oil at pH 3.0 (**a**), pH 4.0 (**b**), and pH 5.5 (**c**) and the temperatures of 25 °C (I), 37 °C (II), and 44 °C (III); colors represent biofilms grown with essential oil (EO) concentration: light blue—without oil, white—0.5 MIC, light green—MIC, dark grey—2.0 MIC. (**d**) PCA analysis of *A. acidoterrestris* biofilm eradication with peppermint oil (factors describe 54.87% total variance); blue, green and red circles represent clusters of strains with similar abilities.

*A. acidoterrestris* strains formed biofilms in the absence of essential oils, regardless of the pH and temperature. However, the level of biofilm formation varied depending on the strain and culture conditions (A_570_ 0.309–2.251) (Figure 1a–c, Figure 2a–c and Figure 3a–c). In general, most environmental isolates formed less biofilms than the reference strain *A. acidoterrestris* DSM 3922 at the optimal temperature for mesophilic microorganisms (25 °C). The level of biofilms of ten isolates (007, 024, 025, 026, 027, 042, 055, 056, 057, and 063) was lower by 9.33–42.35% at pH 3.0 and by 1.79–9.23% at pH 4.0 for six isolates (007, 025, 042, 055, 056, and 057), when compared to the reference strain. Similarly, nine isolates (007, 024, 025, 026, 027, 056, 057, 062, and 063) were observed to form less biofilms at 25 °C at the highest tested pH (pH 5.5) than the DSM 3922 strain. Higher incubation temperatures seemed to promote *A. acidoterrestris* biofilm formation. A significant increase in the environmental strains’ biofilm was found in the cultures incubated at 37 °C or 44 °C at both pH 4.0 and 5.5, with the exception of five strains. Compared to the reference strain, *A. acidoterrestris* 007 formed less biofilm in the culture at pH 3.0 by 11.78% and 4.88% at 37 °C and 44 °C, respectively, and by 4.13% at pH 5.5 (37 °C). Four isolates (024, 025, 026, and 027) produced less biofilms (2.87–38.06%) at 44 °C and pH 5.5 than *A. acidoterrestris* DSM 3922.

All of the essential oils exhibited antibiofilm activity against *A. acidoterrestris;* however, the effect varied depending on the strain and the oil tested. In general, clove oil had a strong effect on *A. acidoterrestris* as the biofilm reduction reached 0.65–100%. Even at the concentration of 0.5 MIC, the oil either inhibited or completely eliminated the biofilm at all analyzed temperatures and pH values (Figure 1a–c). According to the PCA analysis, strains with similar susceptibility to clove oil were grouped into three clusters, with two strains showing a distinct sensitivity (Figure 1d). The biofilms of *A. acidoterrestris* 008 and 062 showed the greatest sensitivity to clove oil in the majority of the variants tested, growing with the increase in the medium acidity. At pH 5.5, the amount of their biofilm reduction varied from 9.71 to 24.52% when the 2 MIC concentration of oil was applied. *A. acidoterrestris* isolates grouped in two closely located clusters (Figure 1d, blue and green clusters) exhibited a relatively similar antibiofilm response to the oils. Environmental isolates 007, 041, and 042 showed high sensitivity to clove oil, especially at 0.5 MIC oil concentration, where a complete biofilm reduction was observed in almost all of the cultures. At pH 3.0 and 4.0, the strains 041 and 042 were less tolerant to the oil than *A. acidoterrestris* 007.

Clove oil eliminated the bacterial biofilm of six strains (009, 024, 025, 026, 027, and 040) at pH 3.0, 4.0, and 5.5 at the concentration of 0.5 MIC, regardless the temperature applied (Figure 1d, green cluster). The weakest oil effect was observed against four environmental strains (055, 056, 057, and 063) and reference *A. acidoterrestris* DMS 3922 (Figure 1d, red cluster). Moreover, increasing the incubation temperature from 25 °C to 37 °C resulted in a greater biofilm reduction.

Based on the sensitivity of the *A. acidoterrestris* biofilms to cinnamon oil, the strains were grouped into three clusters. The largest cluster included nine of the strains tested (007, 026, 040, 041, 042, 056, 057, and DSM 3922) (Figure 2d). *A. acidoterrestris* 024 and 027 appeared to be significantly different from the other bacterial strains, and their biofilms were completely eradicated in the presence of 2 MIC cinnamon oil at pH 3.0 and 4.0. A rather unexpected effect was observed for strain 024, where the addition of essential oil to cultures incubated at 37 °C and pH 3.0 increased the level of biofilm. Most strains (007, 026, 040, 041, 056, 057, 062, 063, and DSM 3922) showed a similar sensitivity to this oil, with their biofilm levels generally being 2.17–59.23% lower than the control samples in most culture variants. A complete biofilm inhibition was noted in the cultures exposed to 2 MIC oil concentration at pH 3.0 at 44 °C (strains 026 and 041) and at pH 4.0 at 25 and 44 °C (strains 007 and 026). *A. acidoterrestris* 008 and 009 were located in a separate cluster due to a moderate resistance to cinnamon bark essential oil (Figure 2d, green cluster). Their biofilms were predominantly eradicated at the 2 MIC oil concentration at all tested pH values at 25 and 37 °C. The average biofilm reduction for strains 025, 042, and 055 ranged from 11.40% to 67.95% regardless of the conditions applied (Figure 2d, blue cluster).

Peppermint oil had the lowest antibiofilm potential against *A. acidoterrestris* of the three oils tested, and no biofilm elimination was observed with any of the culture variants. With the exception of the environmental isolate 007 and the reference strain, the strains were grouped into three separate clusters (Figure 3d). The largest cluster consisted of ten isolates (008, 009, 024, 025, 026, 027, 056, 057, 062, and 063), whose biofilms showed a moderate tolerance to the oil regardless of the pH, temperature, or the oil concentration (Figure 3d, red cluster). The ranges of biofilm inhibition were 5.69–67.37% at pH 3.0, 4.25–57.77% at pH 4.0, and 4.96–41.47% at pH 5.5. Four isolates were paired into two separate clusters due to the slightly different responses of their biofilms to peppermint oil. In general, the reduction in biofilm formation by *A. acidoterrestris* isolates 040 and 041 was between 7.12% and 54.70% under all applied conditions, with the most efficient being at 37 °C (Figure 3d, blue cluster). The antibiofilm action of peppermint essential oil was also observed against strains 042 and 055 (Figure 3d, green cluster) and with biofilm reduction ranging from 3.55% to 61.68%. *A. acidoterrestris* DSM 3922 and isolate 007 were characterized by a high resistance to the oil in the cultures incubated at pH 3.0 and 44.0 °C, though a substantial biofilm reduction was observed in cultures at pH 5.5.

**Figure 4:**
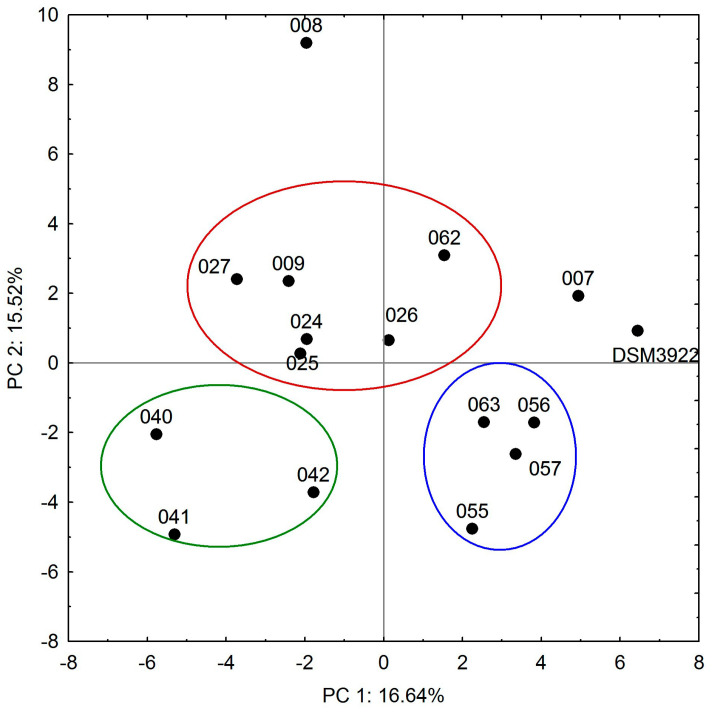
PCA analysis of *A. acidoterrestris* biofilm eradication with clove, cinnamon, and peppermint oils (factors describe 32.16% total variance); blue, green and red circles represent clusters of strains with similar abilities.

The mature biofilms of *A. acidoterrestris* showed a differentiated response to the presence of the three tested essential oils. The clove and cinnamon oils completely eliminated biofilms in several culture variants, which was not observed in the presence of peppermint oil. Taking into consideration the effect of all tested parameters, the results of the PCA analysis grouped the strains into three main clusters, containing six (009, 024, 025, 026, 027, 062), four (055, 056, 057, 063), and three isolates (040, 041, 042) (Figure 4). The remaining strains showed a different response to the oils, but similarities between the reference strain and isolate 007 placed them close together. The growth of biofilms of both strains was strongly affected by 2 MIC cinnamon oil in cultures at pH 3.0, 4.0, and 5.0, predominantly at 25 °C, and by the peppermint oil at pH 3.0 and 5.5 at 25 and 37 °C. Isolates 055, 056, 057, and 063 formed a separate cluster (Figure 4, blue cluster) due to their moderate susceptibility to the cinnamon essential oil, which led to approximately 50% in biofilm reduction in the majority of the conditions tested. Similar results were obtained for clove oil, especially in cultures incubated at a 2 MIC oil concentration mainly at 37.0 and 44.0 °C. The biofilms of six strains in the largest cluster (Figure 4, red cluster) exhibited the most differentiated susceptibility to the oils. Based on all results obtained, the range of essential oil activity against *A. acidoterrestris* biofilms formed by environmental strains is as follows: clove oil > cinnamon oil > peppermint oil.

## 3. Discussion

Microorganisms are known to trigger the mechanisms enabling survival in unfavorable conditions and harsh environments. Such a phenomenon often occurs in bacteria, which exhibit the ability to adhere to surfaces, their colonization, and consequent biofilm formation. The presence of biofilms in the industry is one of major concern, especially when dealing with potential pathogens or extremophiles. The fruit industry is currently struggling with contamination with acidophilic thermophilic bacteria *A. acidoterrestris*. In recent years, considerable research has been conducted worldwide to confirm the ability of these bacteria to colonize various technical surfaces. Podolak et al. [28] stated that *A. acidoterrestris* spores might be able to adhere to stainless steel surfaces. Subsequent studies have proven that the vegetative cells of *A. acidoterrestris* are able to colonize other industrial surfaces used at various stages of production. Apart from stainless steel (SS), adhered alicyclobacilli were observed on glass, polyvinyl chloride (PVC), nylon, polystyrene (PS), and natural food-grade rubber [29,31,32,71,72,73]. In general, many external factors affect the surface colonization process. It is believed that a greater surface roughness and lower medium flux intensity facilitate microbial adhesion. However, in our previous research [31], the majority of the *A. acidoterrestris* strains tested produced more biofilm on a glass surface in agitated cultures than in static ones. A similar tendency was observed in our study on biofilms formed on PVC and glass surfaces [32]. According to Shemesh et al. [30], *A. acidoterrestris* biofilm formation relies on surface motility changes as a response to external pH, linking the increase in medium acidity (from pH 4.5 to below 3.6) with the inhibition of bacteria surface motility and the induction of biofilm formation. Higher levels of *A. acidoterrestris* biofilms were observed at pH 3.0–3.5 than at pH 4.0–4.5, both at the temperatures of 37 and 42 °C. Simultaneously, an induction in biofilm formation was observed at pH 5.5. The results of our study are in agreement with these findings [30]. The majority of the *A. acidoterrestris* strains investigated in our study expressed increased biofilm formation at pH 3.0 compared to pH 4.0 at all temperatures applied (25, 37, 44 °C). Moreover, some strains exhibited an induction in biofilm formation at pH 5.5 predominantly at the temperature favored by mesophilic bacteria. Despite their acidotolerance, *A. acidoterrestris* responds to acidic stress by maintaining intracellular pH homeostasis through a variety of metabolic changes, e.g., enhancing amino acid decarboxylation, urea hydrolysis, and energy supply [74]. Our results suggest that environmental *A. acidoterrestris* isolates may also identify changes in medium acidity as a key factor for either the induction of biofilm formation or the enhancement in their surface motility.

The complex structure of the biofilm provides cells with a strong resistance to environmental stresses [73,75]. Cells in the biofilm are much more resistant to a wide range of stressors, including cleaning agents and disinfectants than their planktonic cells [76]. In the food industry, the control of *A. acidoterrestris* contamination mainly involves heat treatment or adding preservatives or disinfectants [28,29,77], which usually affects the nutritional and sensory value of the final product and may have adverse effects on human health. A promising alternative to sanitizers and disinfectants seems to be antimicrobial substances originating from plants, mainly essential oils that are known to exhibit activity against a wide spectrum of microorganisms [46]. The research presented indicates the antibiofilm potential of cinnamon and clove oils against *A. acidoterrestris* bacteria. Furthermore, experiments involving fifteen environmental isolates under different acidic and thermal stresses exemplify their biofilm-forming activity and the potential for eradication in the presence of substances of natural origin as an alternative to commercial sanitizers.

In our study, *A. acidoterrestris* planktonic cells showed varying, strain-dependent sensitivity to clove, cinnamon, and peppermint essential oils. No literature data were found on the activity of peppermint oil against *A. acidoterrestris*. Few instances in the literature only describe the effects of some essential oils’ components, including cinnamon and clove. Essential oils are mixtures of various components, mainly terpenes and their aerobic derivatives (aldehydes and phenols), which determine their antimicrobial activity. Cinnamon oil is characterized by a high content of (E)-cinnamaldehyde, as well as eugenol, β-caryophyllene, and p-cymene. The cinnamon oil used in this study comprised 72.49% (E)-cinnamaldehyde, 8.12% β-caryophyllene, and 5.17% eugenol. Clove oil mainly consists of eugenol, eugenyl acetate, and β-caryophyllene. Almost 87% of the clove oil used in our research was eugenol. It was found that 25% of the environmental isolates showed a high sensitivity to cinnamon oil, whereas the reference strain *A. acidoterrestris* DSM 3922 was significantly less susceptible. These environmental isolates also exhibited a high sensitivity to clove oil, as did the reference strain. Clove oil in the concentrations used did not affect the growth of the other isolates (MIC ≥ 5.0%). Bevilacqua et al. [78] confirmed the activity of selected cinnamon oil components against *A. acidoterrestris*, indicating that the addition of a mixture of cinnamaldehyde and eugenol effectively ensures the microbiological stability of apple juice. The same research [78] also pointed out the different effect of selected components of essential oils on microbial growth compared with the oils themselves. In the present study, four of fifteen isolates showed high sensitivity to the tested cinnamon oil (MIC ≤ 0.05%) in contrast to the nine isolates with low sensitivity (MIC ≥ 5.0%). Cinnamon oil at a concentration of 3.0% inhibited the growth of the *A. acidoterrestris* DSM 3922 reference strain. In the study by Bevilacqua et al. [78], the growth inhibition of *A. acidoterrestris* was observed at significantly lower concentrations of the active compounds, cinnamaldehyde and eugenol (20–40 ppm and 40–80 ppm, respectively). The lower sensitivity of *A. acidoterrestris* to cinnamon oil may therefore be due to the presence of other chemical compounds that can modulate a destructive effect on the bacterial cell membrane, metabolic pathways, and, as a result, the development of cells and spores. Scientific research is paying more attention to the effects of various plant extracts on *Alicyclobacillus* spp. in fruit juices. The successful prevention of alicyclobacilli growth or spore germination was observed in the presence of methanol-dichloromethane extracts from *Eucalyptus* spp. leaves rich in flavonoids [79], *Piper peltatum* and *P. marginatum* extracts [80], grape seed extract [81], and rosemary extract [82].

Only few reports have evaluated the influence of essential oils on A. *acidoterrestris* towards their biofilms. Our previous research [32] examined the influence of clove essential oil on alicyclobacilli biofilms formed on glass and PVC surfaces under agitated and static conditions. It was found that 500 ppm clove oil reduced the level of biofilms formed on the tested technical surfaces by 25.1–65.0%. Similarly to the findings by Cai et al. [83], clove oil caused morphological changes in *A. acidoterrestris* cells as well as the biofilm structure. The strong properties of this oil may be due to high phenolic compound (eugenol) content, which was previously reported to inhibit alicyclobacilli growth [78]. The present research, which focused on the antibiofilm activity of cinnamon, clove, and peppermint essential oils, confirmed the potential of these oils against *A. acidoterrestris* biofilms on polystyrene, irrespective of the environmental conditions studied. The eradication efficiency of *A. acidoterrestris* biofilms formed on industrial processing equipment by commercial sanitizers strongly depends on the type of surface. According to Anjos et al. [29], the stainless steel and nylon are characterized by a higher level of cell adhesion compared to PVC. Similar studies on stainless steel, wood, and rubber conveyor materials of food contact surfaces indicate a significant variation in the effectiveness of removing *A. acidoterrestris* spores from the surface of different technical materials using industrially applied disinfectants [84]. However, while our studies prove the effectiveness of essential oils against *A. acidoterrestris* biofilms in the laboratory model on a PVC surface, the oils’ antibiofilm efficacy should be verified with the technical materials models, e.g., stainless steel, nylon, or rubber.

The extent to which biofilms were eradicated depended on both the bacterial strain and environmental parameters: acidity, culture temperature, type, and concentration of the essential oil. At the established medium acidity, the growth culture temperature was a factor that influenced biofilm stability more strongly than the type and concentration of the essential oil. On the other hand, at the same culture temperature, *A. acidoterrestris* biofilms showed greater sensitivity to essential oils in media with pH 3.0 and 4.0. The response of biofilms to essential oils varied, with four strains (040, 041, 042, and 055) expressing different sensitivity to cinnamon oil. Clove and cinnamon oils strongly inhibited the development of biofilm of most of the tested strains, regardless of the environment’s acidity and the cultivation temperature.

The strongest antibiofilm activity of clove essential oil may be attributed to eugenol (86.99%), the primary component of the oil, which can affect biofilms by interfering with the integrity of the EPS layer and disrupting the bacterial cell wall, as it was stated in other studies [32,83]. Cinnamon essential oil appeared to exhibit weaker activity towards the tested *A. acidoterrestris* strains than clove oil. Although the oil is mainly composed of cinnamaldehyde (72.49%), which is reported to show strong anti-alicyclobacilli activity [78], the majority of alicyclobacilli biofilms were only reduced by 20–40%. Peppermint essential oil showed the weakest antibiofilm ability. The bacterial strains exhibited differentiated susceptibility to peppermint oil in all the tested culture variants. However, the oil itself did not completely eradicate any alicyclobacilli biofilms.

The antibiofilm efficacy of clove and cinnamon essential oils suggests that they could be used as an efficient alternative to synthetic disinfectants for treating production equipment surfaces. In addition, these oils could be also supplemented directly into fruit juices but in a food-grade amount that does not affect the organoleptic properties (flavor, taste, color, consistency) of the final product. Several studies confirm that combining plant–origin substances with other active substances, or even short-time thermal treatment, can enhance the antimicrobial effect and thus improve the microbial stability of juices [58,59,85]. Therefore, further research is advised on biologically active natural compounds in order to control the development of *A. acidoterrestris* and biofilm formation in the industrial environments.

## 4. Materials and Methods

### 4.1. Microorganisms

The fifteen *A. acidoterrestris* strains used in the study were environmental isolates derived from apple orchards (soil, tree bark, and apples) located in the central region of Poland (Łódź, Poland). The isolates were identified based on 16S RNA gene sequencing (GenBank accession numbers KY045818–KY045832). The reference strain *A. acidoterrestris* DSM 3922 from Deutche Sammlung von Microorganismen und Zellkulturen (Braunschweig, Germany) was included in the study. Bacterial strains were activated according to the procedure described by Tyfa et al. [31]. The bacterial inoculum preparation before each experiment was conducted according to the following procedure. The strains were cultured onto Bacillus acidoterrestris agar pH 4.0 (BAT agar; Merck, Darmstadt, Germany) at 44 °C for 24 h, and their vegetative cells were collected by swabbing and standardized in BAT broth pH 4.0 (Merck, Darmstadt, Germany) to approximately 10^4^ cells/mL using DEN-1 densitometer (Biosan Ltd., Riga, Latvia). The absence of bacterial spores was verified with malachite green staining.

### 4.2. Essential Oils and Their Chemical Analysis

Cinnamon bark (*Cinnamomum zeylanicum* Blume), peppermint (*Mentha piperita* (L.) Huds.), and clove bud (*Syzygium aromaticum* (L.) Merr. & Perry) commercial essential oil were purchased from Pollena Aroma S.A. (Warsaw, Poland). The chemical composition of each oil was determined by gas chromatography-mass spectroscopy (GC-MS) using Trace GC Ultra chromatograph (Thermo Electron Corporation, Waltham, MA, USA) equipped with Rtx-1 (Restek, Bellefonte, PA, USA) non-polar capillary column (60 m × 0.25 mm; 0.25 μm film thickness) combined with DSQ II mass spectrometer (Thermo Electron Corporation, Waltham, MA, USA). The GC-MS procedure was conducted according to Smigielski et al. [86]. The temperature was programmed as follows: 50–300 °C at 4 °C/min; injector (SSL) temperature of 280 °C; the detector (FID) temperature of 300 °C; carrier gas helium with constant pressure of 300 kPa; and split ratio of 1:37. The mass spectrometer operating parameters were: ion source temperature of 200 °C; and ionization energy of 70 eV (EI). The identification of the oil’s components was based on the comparison of their retention indices (RIs), mass spectra (NIST and Wiley Libraries), and the literature [87].

### 4.3. Determination of Antibacterial Activity of Essential Oils

The minimal inhibitory concentration (MIC) of the essential oils was determined by the microdilution method following CLSI recommendations [88]. A total of 20 µL of bacterial inoculum and 100 µL double-concentrated BAT broth were introduced into 1 well of a 96-well polystyrene flat-bottom plates (Corning Inc., New York, NY, USA). Next, the wells were filled with the ethanol–water solution of each essential oil to achieve the final oil concentration. The oils were tested in 10 concentrations in the range of 0.05–5.0% (*v*/*v*). The MIC was considered as the lowest oil concentration at which no visible bacterial growth was observed. The subsequent sub-culturing of the growth cultures with no visible growth onto BAT agar plates (incubation 48 h, 44 °C) was conducted in order to determine the minimal bactericidal concentration (MBC). The oil concentration was assumed as the MBC value for the cultures with a complete absence of bacterial growth on BAT agar. Amoxicillin (25 μg) (BioRad, Warsaw, Poland) was used as a positive control. Each experiment was performed in triplicate.

### 4.4. Bacterial Biofilm Production Assay

The investigation of biofilm production was based on the microtiter plate assay (MTP). Briefly, the wells of a sterile 96-well polystyrene flat-bottom microtiter plate (Corning Inc., New York, NY, USA) were filled with 20 µL of an active bacterial inoculum (10^5^–10^6^ cells/mL) and 180 µL BAT broth (Merck, Darmstadt, Germany) at the selected pH (3.0; 4.0; 5.5) and incubated at the temperatures of 25, 37, or 44 °C for six days. The liquid medium was refilled every 48 h. After six days, the contents of wells were discarded aseptically and 200 µL of fresh medium of particular pH (3.0; 4.0; 5.5) was added to each well. Then, the microtiter plates were incubated at 25, 37, or 44 °C for the next 48 h. After the incubation, the planktonic cells were gently removed and wells were washed three times with 250 µL distilled water. The microtiter plate was left to air dry for 30 min, and then, the adherent bacterial cells were fixed for 20 min with 250 µL methanol solution (Merck, Darmstadt, Germany). Excess of alcohol was discarded, and to quantify the biofilm formation, each well was filled with 250 µL of 0.5% crystal violet solution (Merck, Darmstadt, Germany) and incubated for 15 min at room temperature. Afterwards, the dye solution was discarded and the microtiter plate wells were rinsed three times with distilled water and thoroughly dried at room temperature. The dye staining adherent cells was solubilized with 96% (*v*/*v*) ethanol (Merck, Darmstadt, Germany) for 20 min. The optical density (OD) of each well was measured at 570 nm using microtiter plate reader TriStar2S LB 942 (Berthold Technologies, Bad Wildbad, Germany). To ensure metrological repeatability, each experiment was performed independently in triplicate.

### 4.5. Bacterial Biofilm Production in Presence of Essential Oils

The activity of the selected essential oils on bacterial biofilms was examined using a similar approach to the biofilm production assay. As before, the wells of a sterile 96-well polystyrene microtiter plate were filled with 20 µL of an active bacterial inoculum (10^5^–10^6^ cells/mL) and 180 µL BAT broth of the selected pH (3.0; 4.0; 5.5) and incubated at temperature 25, 37, or 44 °C for six days. The loss of medium was refilled every 48 h. After six days, the liquid content with planktonic cells was removed from each well. In contrast to the biofilm production assay, the wells were filled with 100 µL of concentrated BAT broth (pH 3.0, 4.0, or 5.5) and 100 µL of ethanol–the water essential oils solutions at concentrations of 0.5 MIC, MIC, and 2.0 MIC. MIC values had been previously determined for each essential oil and *A. acidoterrestris* isolate, as it was described in Section 4.3. (Determination of Antibacterial Activity of Essential Oils). The plates were then incubated for 48 h at the temperatures of 25, 37, or 44 °C. Biofilms grown in the presence of essential oils were quantified using a 0.5% crystal violet staining solution (Merck, Darmstadt, Germany) and measured spectrophotometrically using microtiter plate reader TriStar2S LB 942 (Berthold Technologies, Bad Wildbad, Germany), as described above.

### 4.6. Statistical Analysis of Data

Each sample was tested in a triplicate and the average and standard deviations were calculated by means of STATISTICA version 6.0 Pl. Differences between means were statistically calculated using analysis of variance (ANOVA) at *p* ≤ 0.05 significance level. Multiple comparisons between means were made using Tukey’s test. The conditions for biofilm growth in presence of essential oils (bacterial strain, pH, temperature, MICs) were subjected to a principal component analysis (PCA) in the STATISTICA version 6.0 Pl. software (StatSoft Polska LLC, Kraków, Poland).

## 5. Conclusions

The tested commercial clove and cinnamon essential oils expressed substantial antibiofilm activity against *A. acidoterrestris* environmental isolates and the reference strain. The efficiency of alicyclobacilli biofilm eradication depended on both the bacterial strain and growth parameters, such as acidity, culture temperature, type, and concentration of the essential oil used. However, the growth culture temperature had a stronger influence on the biofilms’ stability than the type and concentration of the essential oil in the established medium acidity. Furthermore, at a settled temperature, *A. acidoterrestris* biofilms showed greater sensitivity to essential oils in the medium with lower acidity. Due to its incomplete eradication of any strain biofilm, peppermint oil was classified as the least active of the oils tested. The antibiofilm activity of the essential oils in the study indicated clove and cinnamon essential oils’ potential as effective alternatives to synthetic disinfectants directed against *A. acidoterrestris* growing in the form of biofilms. This may prospectively be used for treating the surfaces of production equipment. Further investigations should be undertaken to concentrate on the oils’ effectiveness against *Alicyclobacillus* biofilms formed on the technical surfaces. The technical material models, e.g., stainless steel, nylon, or rubber, differ in adhesive abilities and should be taken into consideration. Studies should also focus on selected major oil constituents with documented antimicrobial activity. The anti-*A. acidoterrestris* biofilm activity of the main individual components of clove and cinnamon oils should be examined, as their commercial application would significantly reduce the cost with the effect comparable to the one with essential oil.

## Figures and Tables

**Table 1:** Chemical composition of clove, cinnamon, and peppermint essential oils.

Component	Clove Oil		Cinnamon Oil		Peppermint Oil	
	%	RI ^1^	%	RI ^1^	%	RI
α-Pinene	-	-	-	-	0.83	928
Sabinene	-	-	-	-	0.33	963
β-Pinene	-	-	-	-	1.20	967
p-Cymene	-	-	0.11	1009	-	-
1,8-Cineole	-	-	0.28	1017	6.00	1017
Limonene	-	-	0.05	1019	2.03	1019
Linalool	-	-	6.59	1087	-	-
(−)-Menthone	-	-	-	-	22.46	1136
Isomenthone	-	-	-	-	3.31	1143
Menthofuran	-	-	-	-	2.30	1148
(+)-Menthol	-	-	-	-	2.59	1151
(−)-Menthol	-	-	-	-	46.41	1164
Terpinen-4-ol	-	-	-	-	0.43	1170
α-Terpineol	-	-	-	-	0.40	1174
cis-Cinnamaldehyde	-	-	0.24	1181	-	-
Pulegone	-	-	-	-	1.67	1214
Piperitone	-	-	-	-	0.61	1226
trans-Cinnamaldehyde	-	-	72.49	1279	-	-
Menthyl acetate	-	-	-	-	4.01	1577
Eugenol	86.99	1346	5.17	1332	-	-
α-Copaene	0.07	1377	0.15	1375	-	-
trans-Cinnamyl acetate	-	-	4.57	1412	-	-
β-Caryophyllene	8.76	1422	8.12	1421	2.03	1417
α-Humulene	1.91	1452	0.24	1450	0.24	1449
Cadina-1(6),4-diene	0.04	1468	-	-	-	-
γ-Muurolene	0.02	1470	-	-	-	-
Eugenyl acetate	-	-	0.18	1480	-	-
β-Selinene	0.02	1482	-	-	-	-
α-Selinene	0.05	1491	-	-	-	-
β-Farnesene	0.06	1494	-	-	-	-
cis-Calamenene	0.11	1509	-	-	-	-
δ-Cadinene	0.27	1513	-	-	-	-
Cadina-1,4-diene	0.03	1524	-	-	-	-
Caryophyllene oxide	-	-	0.16	1569	-	-
Caryophyllene epoxide	0.62	1571	-	-	-	-
Humulene epoxide	0.11	1594	-	-	-	-
Benzyl benzoate		-	1.05	1723		-
Total	99.06	-	99.40	-	96.85	-

^1^ RI—retention index.

**Table 2:** Antibacterial activity of essential oils against *A. acidoterrestris* planktonic cells grown in a BAT medium (pH = 4.0; 44 °C) expressed as minimum inhibitory concentration (MIC) and minimum bactericidal concentration (MBC) in % *v*/*v*.

Strain	MIC (MBC) (% *v*/*v*)		
	Cinnamon	Clove	Peppermint
DSM 3922	3.0 (5.0)	0.05 (0.06)	0.05 (0.06)
007	>5.0	>5.0	1.0 (1.5)
008	3.0 (5.0)	>5.0	1.5 (3.0)
009	>5.0	>5.0	1.0 (1.5)
024	5.0 (>5.0)	>5.0	1.0 (2.0)
025	>5.0	>5.0	2.0 (3.0)
026	3.0 (>5.0)	>5.0	2.0 (3.0)
027	>5.0	>5.0	3.0 (>5.0)
040	>5.0	>5.0	2.0 (3.0)
041	>5.0	>5.0	1.0 (2.0)
042	>5.0	>5.0	1.5 (3.0)
055	<0.05 (0.05)	0.05 (0.06)	1.5 (3.0)
056	<0.05 (0.05)	0.05 (0.06)	0.05 (0.06)
057	<0.05 (0.05)	0.05 (0.06)	0.05 (0.06)
062	5.0 (>5.0)	(5.0)	1.0 (1.5)
063	<0.05 (0.05)	<0.05 (0.06)	0.06 (0.06)

## Data Availability

Data are contained within the article.
